# Acrylamide Induces Mitophagy and Alters Macrophage Phenotype via Reactive Oxygen Species Generation

**DOI:** 10.3390/ijms22041683

**Published:** 2021-02-08

**Authors:** Chih-Hsing Hung, Yi-Ching Lin, Yi-Giien Tsai, Yu-Chih Lin, Chia-Hong Kuo, Mei-Lan Tsai, Chao-Hung Kuo, Wei-Ting Liao

**Affiliations:** 1 Department of Pediatrics, Kaohsiung Medical University Hospital, Kaohsiung Medical University, Kaohsiung 807, Taiwan; pedhung@gmail.com; 2 Department of Pediatrics, Faculty of Pediatrics, College of Medicine, Kaohsiung Medical University, Kaohsiung 807, Taiwan; snoopy905@gmail.com; 3 Graduate Institute of Medicine, College of Medicine, Kaohsiung Medical University, Kaohsiung 807, Taiwan; 4 Research Center for Environmental Medicine, Kaohsiung Medical University, Kaohsiung 807, Taiwan; 5 Department of Pediatrics, Kaohsiung Municipal Siaogang Hospital, Kaohsiung 812, Taiwan; 6 Department of Laboratory Medicine, Kaohsiung Medical University Hospital, Kaohsiung Medical University, Kaohsiung 807, Taiwan; winterjeanne@gmail.com; 7 Doctoral Degree Program of Toxicology, College of Pharmacy, Kaohsiung Medical University, Kaohsiung 807, Taiwan; 8 Department of Laboratory Medicine, School of Medicine, College of Medicine, Kaohsiung Medical University, Kaohsiung 807, Taiwan; 9 Department of Medical Research, Kaohsiung Medical University Hospital, Kaohsiung Medical University, Kaohsiung 807, Taiwan; 10 Department of Pediatrics, Changhua Christian Children Hospital, Changhua 500, Taiwan; 107239@cch.org.tw; 11 School of Medicine, Kaohsiung Medical University, Kaohsiung 807, Taiwan; 12 School of Medicine, Chung Shan Medical University, Taichung 402, Taiwan; 13 Department of Medical Humanities and Education, School of Medicine, Kaohsiung Medical University, Kaohsiung 807, Taiwan; springfred@gmail.com; 14 Division of Allergology, Immunology and Rheumatology, Department of Internal Medicine, Kaohsiung Medical University, Kaohsiung 807, Taiwan; 15 Department of Biotechnology, College of Life Science, Kaohsiung Medical University, Kaohsiung 807, Taiwan; anson19880325@gmail.com; 16 Department of Internal Medicine, Kaohsiung Municipal Siaogang Hospital, Kaohsiung 812, Taiwan; 17 Division of Gastroenterology, Department of Internal Medicine, Kaohsiung Medical University Hospital, Kaohsiung 807, Taiwan; 18 Department of Medicine, Faculty of Medicine, Kaohsiung Medical University, Kaohsiung 807, Taiwan

**Keywords:** acrylamide, reactive oxygen species (ROS), mitophagy, epigenetics, macrophage

## Abstract

Acrylamide is a readily exposed toxic organic compound due to its formation in many carbohydrate rich foods that are cooked at high temperatures. Excessive production of reactive oxygen species (ROS), which is an important factor for mitophagy, has been reported to lead to airway inflammation, hyper-responsiveness, and remodeling. Epigenetic regulation is an important modification affecting gene transcription. In this study, the effects of acrylamide on ROS productions and mitophagy were investigated. The human monocytic cell line THP-1 was treated with acrylamide, and ROS productions were investigated by flow cytometry. The mitochondrial and epigenetic involvement was evaluated by quantitative real-time PCR. Histone modifications were examined by chromatin immunoprecipitation assays. Mitophagy was detected by Western blotting and confocal laser microscopy. Acrylamide promoted mitochondria-specific ROS generation in macrophages. The gene expression of mitochondrial respiratory chain complex II *SDHA* was increased under acrylamide treatment. Acrylamide induced histone H3K4 and H3K36 tri-methylation in an *SDHA* promoter and increased mitophagy-related PINK1 expression, which promoted a M2-like phenotypic switch with increase TGF-β and CCL2 levels in THP-1 cells. In conclusion, acrylamide induced ROS production through histone tri-methylation in an *SDHA* promoter and further increased the expression of mitophagy-related PINK-1, which was associated with a macrophage M2 polarization shift.

## 1. Introduction

The exposure to air pollution is a significant risk factor for both the development of asthma and the triggering of asthma symptoms [1,2]. Over the past several decades, the prevalence of asthma, allergic disease and atopy has increased significantly in parallel with the increased use of products and materials emitting pollutants, especially volatile organic compounds (VOCs), in the indoor environment [3]. Epidemiological studies suggest an association between exposure to VOCs and adverse allergic and respiratory symptoms of asthma [4,5]. In the pediatric population, the molecular and functional correlation of ambient pollutants with asthma-specific airway inflammation are also highlighted [6,7,8]. The most important sources are cigarette smoking, vehicle-related emissions, building renovation, solvents, household products, and pesticides [9]. VOCs are classified as known or possible irritants, and toxicants, and VOC exposure has been associated with the onset and exacerbation of asthma [3]. Acrylamide is one of the most important VOCs and is carcinogenic, neurotoxic and causes reproductive toxicity when high levels of exposure are reached in mice and rats [10,11]. Humans are also frequently exposed to acrylamide due to its formation in many carbohydrate rich foods cooked at high temperatures [12]. Asthma is associated with an increase in endogenous reactive oxygen species (ROS) formation, leading to oxidative stress-induced damage to the respiratory system [13,14]. Environmental antigen exposure has been demonstrated to overproduce ROS, resulting in abnormal physiologic function of DNA, proteins, and lipids that can augment bronchial hyperresponsiveness and inflammation in clinical settings. Oxidative stress has been considered to be important in the pathogenesis of allergic asthma in animal and human studies. Redox mechanisms of oxidative stress play a key role in the pathogenesis of asthma and represent a promising therapeutic target. [15]. ROS, which are products of normal cellular metabolism and act as second messengers, participate in maintenance of cellular ‘redox homeostasis’ to protect cells against oxidative stress under physiological conditions. Regulation of redox states is important for cell activation, viability, proliferation, and organ function. Overproduction of ROS, most frequently because of excessive stimulation of either reduced nicotinamide adenine dinucleotide phosphate (NADPH) by pro-inflammatory cytokines, such as tumor necrosis factor-α (TNF-α) and interleukin-1β (IL-1β) or the mitochondrial electron transport chain, results in oxidative stress [16]. Oxidative stress is a deleterious process that leads to airway and lung damage, and consequently to several respiratory inflammatory injuries, including acute respiratory distress syndrome (ARDS), asthma and chronic obstructive pulmonary disease [17,18,19]. Therefore, oxidative stress regulates both key inflammatory signal transduction pathways and target proteins involved in airway and lung inflammation.

Epigenetic regulations, including histone acetylation or methylation, are important modifications affecting gene transcription and are controlled by the action of histone acetyltransferase (HAT), deacetylase (HDAC) and methyltransferase. Chromatin carries numerous histones associated with gene transcription and modifications on histones, such as acetylation or trimethylation at H3K4, H3K36 and H3K79, and are associated with gene activation [20]. Mitophagy, or autophagy of the mitochondria, is important for mitochondrial quality control and is thereby essential for providing cellular energy, calcium homeostasis, redox signaling and apoptotic signaling [21].

An association between exposure to acrylamide and adverse effects on asthma are demonstrated in some studies. However, the detailed mechanisms of the adverse effect of acrylamide on asthma development remain unclear. ROS and subsequent mitophagy play critical roles in the pathogenesis of asthma. In the present study, we investigated the effects of acrylamide on ROS production and the subsequent mitophagy.

## 2. Results

### 2.1. Acrylamide Induced Mitochondria Associated ROS Generation

**Figure 1:**
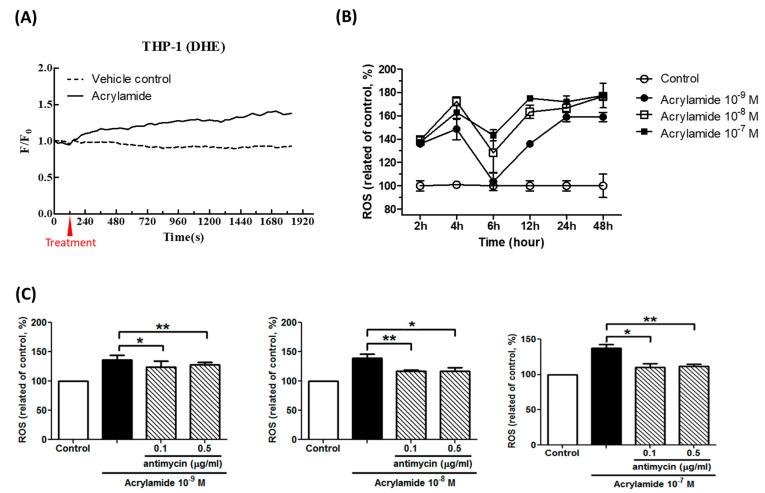
Acrylamide induced reactive oxygen species (ROS) production in THP-1 cells. (**A**) Acrylamide (10^−9^ M) immediately induced ROS in THP-1 cells. The ROS was detected by dihydroethidium (DHE) staining. (**B**) The pre-treatment of acrylamide (10^−9^ M~10^−7^ M) significantly induced ROS expression in THP-1 cells at 2, 4, 12, 24 and 48-h, but less in 6-h. (**C**) The pre-treatment of antimycin A, a complex III of respiratory chain inhibitor, suppressed acrylamide-induced ROS generation in THP-1 cells in 2 h. * *p* < 0.05, ** *p* < 0.01, by the Kruskal-Wallis test; *n* = 9, means ± SD.

Using mitochondria-specific ROS detection dye dihydroethidium (DHE) and general ROS detection dye dichlorodihydrofluorescein (DCFH), we tested the effects of acrylamide on ROS generation. The result showed that acrylamide treatment immediately and significantly increased DHE signal between 0–30 min in THP-1 cells (Figure 1A). This increased ROS (by DHE staining) between 2 and 4 h but reversed at 6 h after treatment (Figure 1B). We selected 2 h as a detection time point and found that acrylamide-induced ROS was able to be inhibited by mitochondria respiratory chain inhibitor antimycin A treatment (Figure 1C).

### 2.2. Mitochondrial Respiratory Chain Complex II Is a Target for Acrylamide

**Figure 2:**
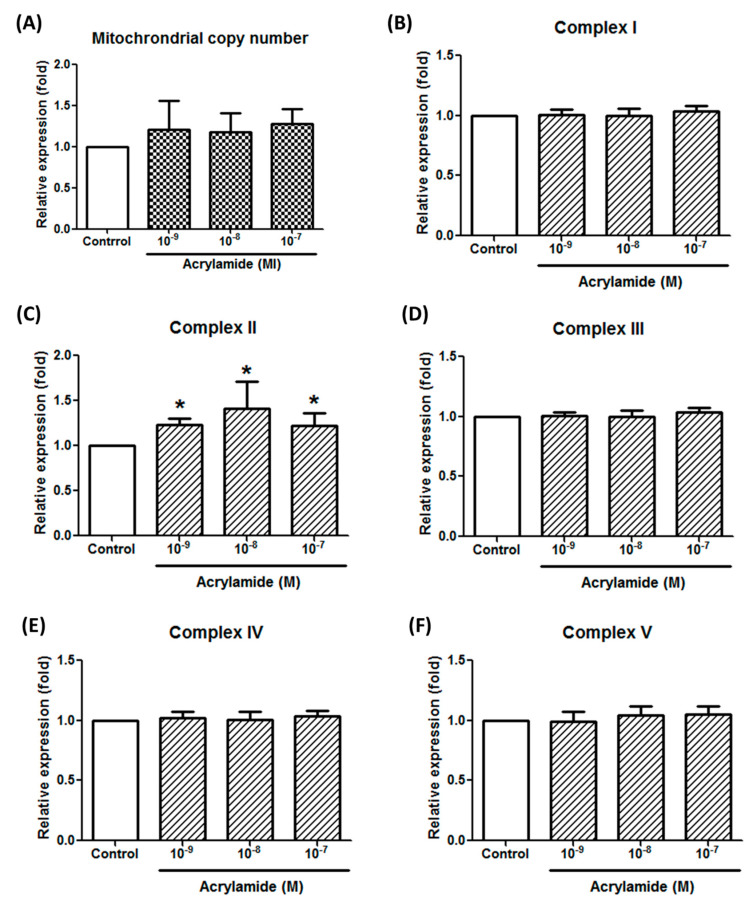
Acrylamide-induced respiratory chain complex II gene expression in THP-1 cells. (**A**) Mitochondrial copy number in THP-1 cells were calculated by detecting the ratio of mitochondria-specific *ND-1* gene expression and chromosome-specific *18S-rRNA* gene expression by real-time PCR. (**B**) The effects of acrylamide on mitochondrial respiratory chain complex I specific gene *ND-1* expression were detected by real-time PCR. (**C**–**F**) The effects of acrylamide on the expressions of mitochondrial respiratory chain complex II specific gene *SHDA*, complex III gene *CYTB*, complex IV gene *COX-1* and complex V gene *ATP6*, were detected by real-time PCR. Among detected mitochondrial complex genes, the complex II specific gene *SHDA* was significantly increased by acrylamide treatments. * *p* < 0.05 by the Kruskal-Wallis test; *n* = 6, means ± SD.

The inhibition of acrylamide-induced ROS expression by antimycin A suggested mitochondrial involvement. Next, we tried to evaluate the involvement of respiratory chains, including complex I to V and uncoupling protein. To investigate acrylamide-induced mitochondrial changes, we detected the mitochondrial copy number and complex I–V gene expressions. Acrylamide treatment (2 h) did not significantly change mitochondria copy number (NS-1/18s ratio) (Figure 2A). By screening respiratory chain complex I gene *ND1*, complex II *SDHA*, complex III *CYTB*, complex IV *COX1* and complex V *ATP6*, we found that acrylamide significantly increased complex II *SDHA* gene expression, but not others (Figure 2B–F).

### 2.3. Acrylamide Promoted Complex II SDHA Expression via Epigenetic Histone Modification

**Figure 3:**
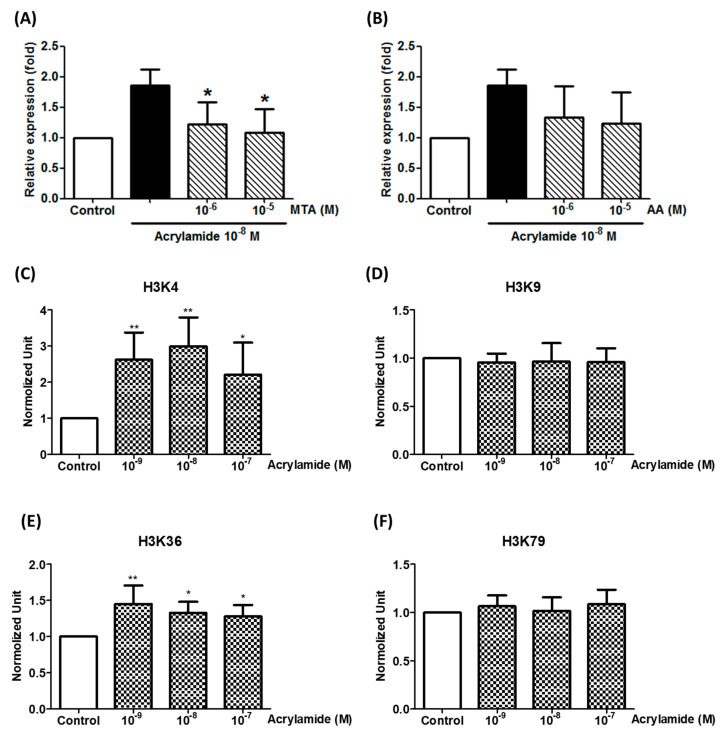
Acrylamide-induced SDHA expression regulated by histone trimethylation. (**A**) The expression of SHDA was detected by real-time PCR after acrylamide (10^−8^ M) and histone acetyltransferase inhibitor anacardic acid (AA) co-treatments (2 h). (**B**) The expression of SHDA was detected by real-time PCR after acrylamide (10^−8^ M) and histone methyltransferase inhibitor 5’-methylthioadenosine (MTA) co-treatments (2 h). * *p* < 0.05, ** *p* < 0.01, by the Kruskal-Wallis test; *n* = 6, means ± SD. (**C**–**F**) The effects of acrylamide on H3K4, H3K9, H3K36 and H3K79 trimethylation of the SHDA promoter was analyzed by ChIP assays. * *p* < 0.05, ** *p* < 0.01, by the Kruskal-Wallis test; *n* = 6, means ± SD.

We further studied the epigenetic status in the *SDHA* promoter to explore the mechanism of acrylamide-induced *SDHA* expression. The data showed that acrylamide-induced SDHA expression was able to be inhibited by histone methyltransferase in inhibitor MTA pre-treatment (Figure 3A), but not histone acetyltransferase anacardic acid (AA) pretreatment (Figure 3B). This data indicated that histone methylation was involved in acrylamide-induced *SDHA* expression. Further, we studied the trimethylation sites in H3 and found that acrylamide increased H3K4 and H3K36 methylation in *SDHA* promoter, but not H3K9 and H3K79 (Figure 3C–F).

### 2.4. Acrylamide Induced Mitophagy-Related Protein Expression

**Figure 4:**
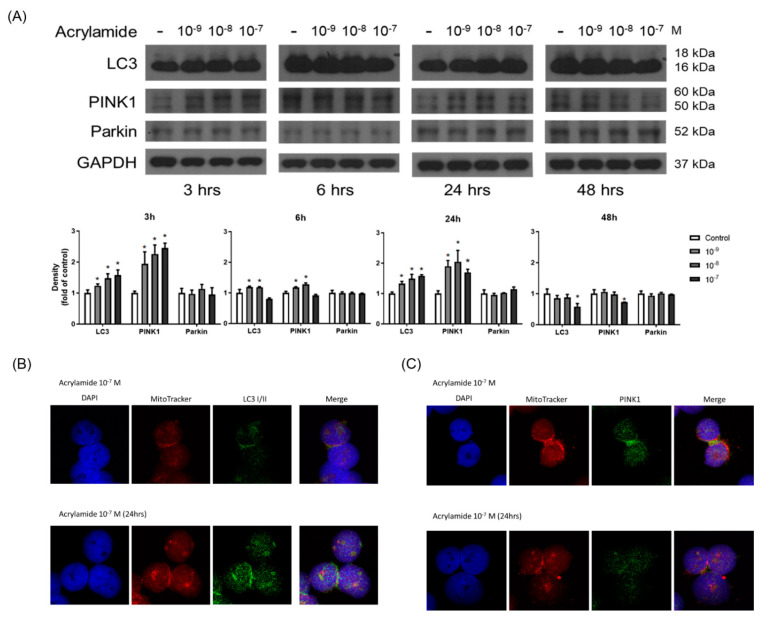
Acrylamide induced mitophagy in THP-1 cells. (**A**) Western blotting of LC3, PINK1, and Parkin in acrylamide-treated THP-1 cells (*n* = 4). (**B**,**C**) By immunofluorescence staining, the co-localization mitochondria (by mito-tracker; red) and LC3 or Parkin (green) were tested (*n* = 3). * *p* < 0.05, by the Kruskal-Wallis test.

Acrylamide significantly induced ROS production and ROS plays an important role in the mitophagy. We further examined whether acrylamide could also induce mitophagy-related protein expression in human monocytes. Western blot showed that acrylamide (10^−7^–10^−9^ M) significantly increased expressions of mitophagy marker PINK1, but not Parkin, and autophagy marker LC3 expression at 3 h treatment time point. The enhancement in PINK1 and LC3 continued to 6 and 24 h, but eased at 48 h. (Figure 4A). These changes were confirmed by PINK1 and LC3 staining via confocal laser microscopic analysis (Figure 4B,C).

### 2.5. Acrylamide-Induced Mitophagy Was Associated with Macrophage M2 Polarization Shift

**Figure 5:**
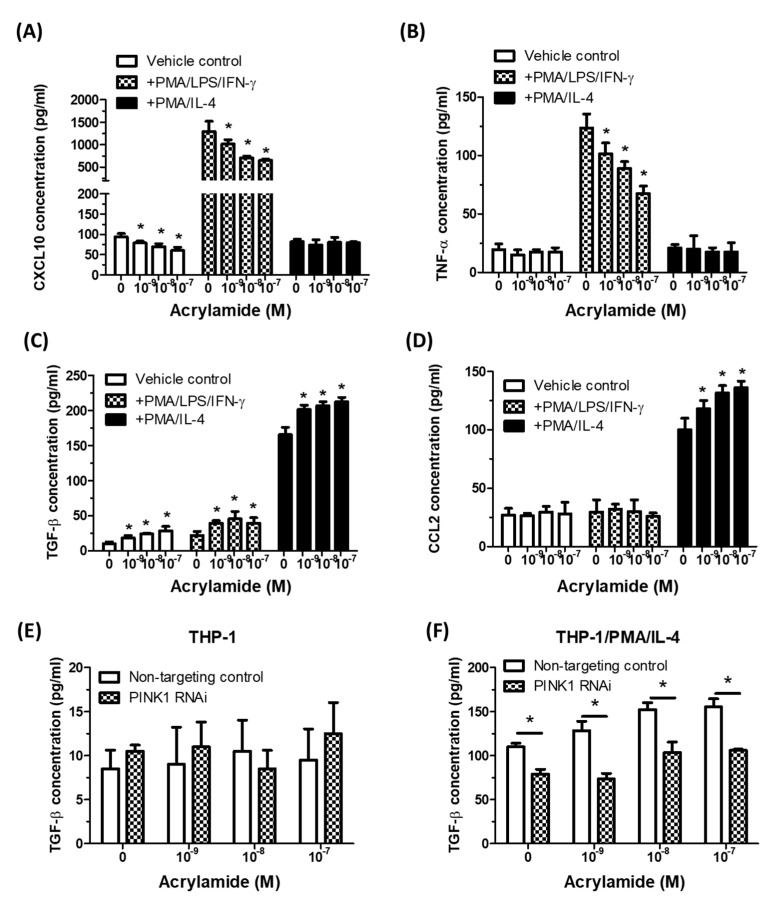
Acrylamide-induced mitophagy was associated with macrophage M2 polarization shift. (**A**,**B**) The tested THP-1 cells were pretreated with acrylamide and exposed to PMA/LPS/IFN-γ that priming M1 polarization, or exposed to PMA/IL-4 that primed M2 polarization. After 24 h, the cell-free medium were collected and macrophage M1 polarization markers CXCL10 and TNF-α were tested by ELISA. (**C**,**D**) THP-1 cells were pretreated with acrylamide, with or without PMA/LPS/IFN-γ or PMA/IL-4 treatments. After 24 h, the cell free medium were collected and macrophage M2 polarization markers TGF-β and CCL2 were tested by ELISA. (**E**,**F**) TGF-β concentrations in THP-1 and PMA/IL-4 primed THP-1 (24 h after acrylamide treatments) with or without PAKIN1 RNAi were detected by ELISA. * *p* < 0.05, by the Kruskal-Wallis test; *n* = 3, means ± SD.

To evaluate the functional outcomes in acrylamide-induced monocyte alterations, we tested the effects of acrylamide treatments on macrophage M1/M2 polarization using PMA/LPS/IFN-γ primed THP-1 (toward M1 phenotype) and PMA/IL-4 primed THP-1 cells (toward M2 phenotype). The results showed that acrylamide treatments significantly decreased the release of M1 markers CXCL10 and TNF-α in PMA/LPS/IFN-γ primed THP-1 cells (Figure 5A,B). In parallel, acrylamide treatments significantly increased the release of M2 markers TGF-β and CCL2 in PMA/IL-4 primed THP-1 cells (Figure 5C,D). Furthermore, this acrylamide-induced M2 shift was suppressed by PINK1 gene knockdown (Figure 5E,F), indicating acrylamide-induced mitophagy could promote macrophage M2 polarization shift.

## 3. Discussion

Our study provided two significant new insights in biological effects of a common environmental/dietary pollutant, acrylamide. Functionally, we found that acrylamide induced a M2 phenotypic switch in human monocytic immune cells. Mechanistically, we identified the regulation from acrylamide-induced histone modification in mitochondrial complex SHDA gene mediated ROS generation, and mitophagy. This acrylamide-induced mitophagy can regulate a M2 phenotypic switch in LPS or IL-4 primed monocytes. Since M2 macrophages play critical roles in multiple pathologic processes such as asthma and carcinogenesis, these findings provide an evidence-based impact on human health issues of acrylamide exposure.

The adverse effects of rising environmental pollution have been of increasing concern in industrializing nations with rising urbanization patterns. Worldwide trends of asthma prevalence are also provided which detail the prominent rise in asthma symptoms in many urban areas. The correlation of motor vehicle, industrial and cooking energy sources are the major emitters among the pollutants in urban settings, with asthma epidemiology in children [6,22]. Several mechanisms contribute to this risk because a wide range of deleterious air pollutants contribute to the pathogenesis of asthma, including altered innate and adaptive immune responses, gene–environment interactions, epigenetic regulation, and possibly gene–environment–epigene interactions [23,24,25].

Asthma is a complex disease with variant etiologies and there is effective correlation between the concentration of VOCs and asthma severity and control [26]. Acrylamide, one of the VOCs, is a synthetic chemical compound commonly used in the synthesis of polyacrylamides, which are widely employed in plastics, paints, varnishes, adhesives and mortars production and is known as a neurotoxin in humans [27]. Acrylamide is also a toxin that humans are readily exposed to due to its formation in many carbohydrate-rich foods cooked at high temperatures. Acrylamide is carcinogenic, neurotoxic and causes reproductive toxicity when high levels of exposure are reached in mice and rats. Acrylamide was first detected in food in 2002, and studies on acrylamide analysis, occurrence, formation, toxicity, risk assessment and mitigation have been extensively carried out and the presence of acrylamide in lots of fried and baked foods raises concerns due to its potential to cause toxicity and cancer in animals and humans [28,29,30].

ROS are important mediators of natural physiological processes. Inflammatory cells recruited to the asthmatic airways have an exceptional capacity for producing a variety of highly reactive ROS believed to contribute to tissue damage and chronic airway inflammation in asthma [31]. In addition, the use of redox-based therapy to attenuate levels of ROS presents a prevention strategy to alleviate oxidative stress-induced airway inflammation in patients with asthma [32]. In the present study, acrylamide induced obvious ROS production, which is an important factor in pathophysiology of asthma (Figure 1A,B). This finding may link the relationship between acrylamide exposure and asthma. Acrylamide induced obvious ROS production in THP-1 cells, however, the detailed mechanisms are still unclear. In the present study, we evaluated the mechanism underlying acrylamide-induced ROS production. Antimycin A, a respiratory chain III inhibitor, interrupted the electron transfer from the cytochrome b to the Qi-site, therefore, disrupted the mitochondrial Q-cycle of enzyme turnover [33,34,35]. Antimycin A effectively suppressed acrylamide-induced ROS production, suggesting that respiratory chain signaling is the major source of arylamide-induced ROS (Figure 1C). Thus, our research suggests that the assessment of oxidative stress byproducts represents one of the mechanisms induced by VOC. ROS production by mitochondria involves the change mitochondria copy number and respiratory chain complex I-V. Our results of screening respiratory chain complex I gene *ND1*, complex II *SDHA*, complex III *CYTB*, complex IV *COX1* and complex V *ATP6* suggested that acrylamide significantly increases complex II *SDHA* gene expression, but not others (Figure 2).

Another important finding of this study is that we provided additional insight into the epigenetic regulation of VOC-induced ROS production on immune cells. Modifications on histones, such as acetylation or trimethylation at H3K4, H3K36, and H3K79, are associated with gene activation, and these modifications are usually carried out by a variety of histone acetyltransferases or methyltransferases [36]. In the present study, we found that acrylamide could increase H4 acetylation and H3K4 and H3K36 trimethylation also in the *SDHA* gene promoter area. This evidence suggested that epigenetic regulation may be one of the important mechanisms by which VOC induced ROS production.

Mitochondria are essential organelles for several fundamental cellular processes, including energy production, metabolite synthesis, iron and calcium homeostasis, and programmed cell death. Mitochondrial quality influences not only individual cell functions but also whole-body metabolism. Mitochondria are a critical source of ROS and dysregulated mitochondrial quality control is associated with promoting TGF-β activity that contributes to airway remodeling in allergic asthma [37]. Therefore, mitochondrial-targeted antioxidants may be a novel approach for future asthma [38]. Mitophagy, which is essential for providing cellular energy and redox, is emerging as a key quality-control mechanism in mitochondria [21]. Mitophagy is also the selective degradation of mitochondria by autophagy occurring to defective mitochondria following damage or stress, e.g., massive ROS load and depolarization [39]. Another key pathogenesis of asthma is airway remodeling with pulmonary fibrosis in which TGF-β1 plays an important role. TGF-β1 induces lung epithelial cell mitochondrial ROS and depolarization, and stabilizes the key mitophagy initiating protein, PINK1. PINK1 ameliorates epithelial cell death and may be necessary to limit fibrogenesis [40]. Recently, Akt1-mediated ROS production and mitophagy contributes to alveolar macrophage apoptosis resistance and is required for pulmonary fibrosis [41]. In the present study, acrylamide obviously induced ROS production and subsequently increased mitophagy-associated protein PINK1 production. Therefore, the exposure to acrylamide may induce the ROS production and mitophagy of monocyte-macrophage, which may be associated with pulmonary fibrosis in asthma.

Several recent studies suggested that mitophagy regulates macrophage phenotypes [42,43]. For example, mitophagy significantly blocks high-glucose induced M1 makers (iNOS and TNF-α) expression and enhances M2 makers (MR and Arg-1) expression in rat macrophages [42]. In the present study, acrylamide promoted macrophage polarization toward a M2 phenotype. Similar M2 promoting effects by acrylamide-induced mitophagy in human monocyte cell line was found. At least in part, our data suggested that TGF-β is one of the increased M2 factors by acrylamide-induced mitophagy. Recently, we identified an aberrant differentiation of monocytes into M2-like cell subsets and fibrocytes, together with increased monocyte-derived TGF-β1, and characterized patients with severe asthma [44]. To correlate the level of acrylamide exposure and blood TGF-β1 in the asthma patients would be our future study topic to clarify acrylamide related health effects.

The detailed mechanisms of acrylamide-associated carcinogenic effects are still unclear. In the present study, acrylamide induced obvious ROS production and subsequent mitophagy. Mitophagy is emerging as a key quality control mechanism in cancer cells. Malignant transformation is found to induce marked changes in mitochondrial dynamics and structure [45]. One of the key hallmarks of tumor progression is metabolic reprogramming, which deregulates ROS content and renders cells more susceptible to mitochondrial perturbations [46]. Despite mitophagy increasing relevance in cancer biology, the field of acrylamide-induced mitophagy remains virtually unexplored in cancer. The understanding of the mechanisms that underlie the effect of exposure to acrylamide on cancer diseases can help identify targets for therapy. These interventions include pollutant source reduction among those most exposed and most vulnerable, and novel pharmaceutical strategies to reduce asthma and cancer morbidity. Both high quality, robust observational studies and ultimately clinical trials are needed to assess the impact of interventions that aim to reduce VOC exposure in patients with asthma and cancer.

## 4. Materials and Methods

### 4.1. Cell Preparation and the Measurement of ROS Production

The human monocytic cell line, THP-1 (American Type Culture Collection, Rockville, MD), was cultured in RPMI-1640 medium (Gibco, Carlsbad, CA, USA) with 10% fetal bovine serum (Gibco, Carlsbad, CA, USA), 100 U/mL penicillin, 100 µg/mL streptomycin and 0.25 µg/mL amphotericin B at 37 °C and 5% CO_2_ in a humidified incubator. The cells were resuspended in fresh media at a concentration of 1 × 10^5^/mL, 1 mL/well in 24 wells plates or 1 × 10^6^/mL, 2 mL/well in 6 well plates overnight for attachment, and 10 mcg/mL of poly I:C (Sigma Chemical, MO, USA) was added with or without pretreatment of the cells with acrylamide for different time points. ROS production was measured with DCFH-DA by a flow cytometry [47]. DCFH-DA is cleaved intra-cellularly by nonspecific esterases and turned to highly fluorescent 20,70-dichlorofluorescin (DCF) upon oxidation by ROS. Following treatment of anti-asthmatic medications, the medium was aspirated and cells were incubated with 1 mM MDCFH-DA in Phosphate-buffered saline (PBS) at 37 °C for 15 min, then cells were washed twice with serum-free medium. For flow cytometry assay, THP-1 cells were probed with 1 mM DCFH-DA and then washed with PBS once for immediate dichlorofluorescein (DCF) detection. The generation of ROS was measured using DCF-HDA, an oxidation-sensitive fluorescent probe. Intracellular H_2_O_2_ or low molecular weight peroxides can oxidize DCFH-DA to the highly fluorescent compound DCF. Signals were detected through a 525 nm band-pass filter (FL1 channel) using a FACS Calibur flow cytometer (Becton Dickinson, San Jose, CA, USA) [47]. To investigate the mitochondria involvement in acrylamide-related macrophage polarization, the cells were pretreated with antimycin A (0.5 μg/mL, Sigma-Aldrich, St. Louis, MO, USA) 1 h before acrylamide treatment [48]. To investigate epigenetic regulation, the cells were pretreated with methylthioadenosine (MTA, a histone methyltransferase inhibitor) or anacardic acid (AA, a histone acetyltransferase inhibitor) 1 h before acrylamide treatment [49].

### 4.2. Quantitative Real-Time PCR (qRT-PCR) for Respiratory Chains and Mitochondrial Involvement

Total RNA (1 μg) was reverse transcribed by SuperScript II using an anchored oligo-dT primer as described by the manufacturer (Invitrogen, Carlsbad, CA, USA). The resulting DNA was used as a template for qRT-PCR. Primer pairs were designed using Primer3 (http://frodo.wi.mit.edu/primer3/ (accessed on 6 February 2021)). Primer pairs were validated using in-silico PCR (http://genome.ucsc.edu/cgi-bin/hgPcr (accessed on 6 February 2021)) and BLAST (http://blast.ncbi.nlm.nih.gov/Blast.cgi (accessed on 6 February 2021)) as the following primer sequence: *MT-ND1*: NADH dehydrogenase, subunit 1 (MT complex I) Forward: 5′-ACCATTTGCAGACGCCATAA, Reverse: 5′-TGAAATTGTTTGGGCTACGG; *SDHA*: succinate dehydrogenase complex, subunit A, flavoprotein (MT complex II): Forward: 5′-CAAACAGGAACCCGAGGTTTT, Reverse: 5′-CAGCTTGGTAACACATGCTGTAT; *MT-CYTB*: mitochondrial cytochrome b (MT complex III): Forward: 5′-GCCCTCGGCTTACTTCTCTT, Reverse: 5′-GACGGATCGGAGAATTGTGT; *COXI* (MT-COI): cytochrome c oxidase I (MT complex IV): Forward: 5′-TTCGCCGACCGTTGACTATTCTCT, Reverse: 5′-AAGATTATTACAAATGCATGGGC; *MT-ATP6*: ATP synthase, H+ transporting, mitochondrial Fo complex, subunit F6 (MT complex V): Forward: 5′-TTTGCGGAGGAACATTGGTGT, Reverse: 5′-TCCAGATGTCTGTCGCTTAGAT; *UCP2*: uncoupling protein 2 (mitochondrial, proton carrier): Forward: 5′-CCTGAAAGCCAACCTCATGAC, Reverse: 5′-CAATGACGGTGGTGCAGAAG; *18 S rRNA*: Forward: 5′-TAGAGGGACAAGTGGCGTTC, Reverse: 5′-CGCTGAGCCAGTCAGTGT.

For qRT-PCR time course samples (*n* = 3), 10 μL reactions consisting of 3 μL diluted cDNA and 0.3 μM of forward and reverse gene-specific primers combined with 2 × Power SYBR Green PCR Master Mix (Applied Biosystems, Foster City, CA) were aliquoted into 96-well plates using a Biomeck^®^ 2000 Laboratory Automation Workstation (Beckman Coulter Inc., Fullerton, CA, USA). Amplification was conducted on an Applied Biosystems PRISM 7900HT Sequence Detection System consisting of a 95 °C denaturation stage for 10 min, then 40 repetitions of 95 °C for 15 s, followed by 60 °C for 1 min. Quantification was determined using the comparative CT method (ΔΔCT) (Applied Biosystems, Foster City, CA, USA). The geometric mean of housekeeping gene (GAPDH) expression was used as the internal control. The relative copy number of mtDNA was measured by normalization of the crossing points in quantitative PCR curves between the mitochondrial *ND1* gene and the nuclear *18S rRNA* gene, and the ratio was normalized to the control [50].

### 4.3. Western Blotting

After treatment for different time points of acrylamide, THP-1 cells were lysed with equal volumes of ice-cold 150-µL lysis buffer 1 h later. After centrifugation at 13,000× *g* for 15 min, equal amounts of cell lysates were blotted and analyzed by western blot with anti-LC3 (MBL, Nagoya, Japan), anti-PINK1 (Cell Signaling Technology, Danvers, MA, USA), anti-parkin and GADPH antibodies (Santa Cruz Biotechnology, Santa Cruz, CA, USA). Immunoreactive bands were visualized by using horseradish peroxidase-conjugated secondary antibody and the enhanced chemiluminescence (ECL) system (Amersham Pharmacia Biotech). The assay was performed by using the protocols recommended by the manufacturer.

### 4.4. Chromatin Immunoprecipitation Assay (ChIP)

5 × 10^5^ cells were treated with 1% formaldehyde for 10 min at room temperature, followed by sonication of DNAs and immunoprecipitation of chromatins overnight with antibodies for acetylated H3 and H4, and trimethylated H3K4 (Upstate Biotechnology, Waltham, MA, USA). Immune complexes were collected using a protein A slurry (Invitrogen, Carlsbad, CA, USA), and the DNA was reverse cross-linked, extracted, and quantified on a Taqman SDS 7900HT. All Taqman reagents were purchased from Applied Biosystems. The relative intensities of the amplified products were normalized to the input DNAs.

DNA from immune complex, by anti-acetylated H3 and H4 and trimethylated H3K4, H3K36, and H3K79 antibodies, were quantified using designed primer for *SDHA* promoter regions encompassing the following subregions relative to the transcription start sites: *SDHA* (-2975/-2577, including AP-1 binding site) according to the predicted of MatInspector (Genomatix Software; München, Germany).

SDHA -2975/-2577 primer sequence: Forward: 5′- ctgaccacactacctcagca; Reverse: 5′- cagtgcttgcttcttggtga.

### 4.5. Immunofluorescent Staining

For immunofluorescent staining, the cells were fixed on a glass slide and co-stained with Mito Tracker (red) and FITC-conjugated antibody against LC3 or PINK1. These cells were then stained with DAPI as DNA counter staining and mounted for observation under a fluorescence microscope (LSM Fluoview 500, Olympus).

### 4.6. PINK1 Knockdown

Using the siGENOME SMARTpool system, human PINK1 siRNA was chemically synthesized and one tube contained a mixture of SMART selection-designed siRNA targeting gene for in-vitro transfection. By the same way, the siGENOME non-targeting siRNA pool was also performed (Thermo, Dharmacon, Inc. DBA). THP-1 cells were seeded into 24-well culture plates and transfected with 100 nM of siRNA per well using Xfect^TM^ siRNA transfection reagent (Clontech, CA, USA) according to the manufacturer’s instruction. Cells were treated (acrylamide/PMA/IL-4) at 24 h after transfected and the outcomes of TGF-β release were detected at 48 h after transfection.

### 4.7. Statistical Analysis

Statistical analysis was performed using IBM SPSS Statistics (Version 19, IBM Company, Armonk, NY, USA) and GraphPad Prism (Version 5, GraphPad Prism Software, Los Angeles, CA, USA). All values are presented as means ± SDs. Nonparametric statistical tests were used in this study. All values are presented as means ± standard deviations (SDs). Comparisons among multiple groups were assessed by the Kruskal-Wallis test followed by Dunn’s post-test as appropriate. A *p*-value of < 0.05 was considered statistically significant.

## Data Availability

The data presented in this study are available on request from the corresponding author. Data may be available upon request to interested researchers. Please send data requests to: Wei-Ting Liao, PhD. Department of Biotechnology, College of Life Science, Kaohsiung Medical University.

## References

[1] Lee S.-Y., Chang Y.-S., Cho S.-H. (2013). Allergic diseases and air pollution. Asia Pac. Allergy.

[2] Jacquemin B., Kauffmann F., Pin I., Le Moual N., Bousquet J., Gormand F., Just J., Nadif R., Pison C., Vervloet D. (2012). Air pollution and asthma control in the Epidemiological study on the Genetics and Environment of Asthma. J. Epidemiol. Community Health.

[3] Tagiyeva N., Sheikh A. (2014). Domestic exposure to volatile organic compounds in relation to asthma and allergy in children and adults. Expert Rev. Clin. Immunol..

[4] Rive S., Hulin M., Baiz N., Hassani Y., Kigninlman H., Toloba Y., Caillaud D., Annesi-Maesano I. (2013). Urinary S -PMA related to indoor benzene and asthma in children. Inhal. Toxicol..

[5] Nurmatov U.B., Tagiyeva N., Semple S., Devereux G., Sheikh A. (2015). Volatile organic compounds and risk of asthma and allergy: A systematic review. Eur. Respir. Rev..

[6] Jassal M.S. (2015). Pediatric asthma and ambient pollutant levels in industrializing nations. Int. Health.

[7] Moerloose K.B., Robays L.J., Maes T., Brusselle G., Tournoy K.G., Joos G.F. (2006). Cigarette smoke exposure facilitates allergic sensitization in mice. Respir. Res..

[8] Manners S., Alam R., Schwartz D.A., Gorska M.M. (2014). A mouse model links asthma susceptibility to prenatal exposure to diesel exhaust. J. Allergy Clin. Immunol..

[9] Chin J.-Y., Godwin C., Parker E., Robins T., Lewis T., Harbin P., Batterman S.A. (2014). Levels and sources of volatile organic compounds in homes of children with asthma. Indoor Air.

[10] El-Sayyad H.I., El-Gammal H.L., Habak L.A., Abdel-Galil H.M., Fernando A., Gaur R.L., Ouhtit A. (2011). Structural and ultrastructural evidence of neurotoxic effects of fried potato chips on rat postnatal development. Nutrition.

[11] National Toxicology Programme (2012). Toxicology and carcinogenesis studies of acrylamide (CASRN 79-06-1) in F344/N rats and B6C3F1 mice (feed and drinking water studies). Natl. Toxicol. Program. Tech. Rep. Ser..

[12] Pan X., Guo X., Xiong F., Cheng G., Lu Q., Yan H. (2015). Acrylamide increases dopamine levels by affecting dopamine transport and metabolism related genes in the striatal dopaminergic system. Toxicol. Lett..

[13] Dvash E., Har-Tal M., Barak S., Meir O., Rubinstein M. (2015). Leukotriene C4 is the major trigger of stress-induced oxidative DNA damage. Nat. Commun..

[14] Sahiner U.M., Birben E., Erzurum S., Sackesen C., Kalayci Ö. (2011). Oxidative stress in asthma. World Allergy Organ. J..

[15] Jiang L., Diaz P.T., Best T.M., Stimpfl J.N., He F., Zuo L. (2014). Molecular characterization of redox mechanisms in allergic asthma. Ann. Allergy Asthma Immunol..

[16] Nadeem A., Siddiqui N., Alharbi N.O., Alharbi M.M. (2014). Airway and systemic oxidant-antioxidant dysregulation in asthma: A possible scenario of oxidants spill over from lung into blood. Pulm. Pharmacol. Ther..

[17] Lee I.-T., Yang C.-M. (2012). Role of NADPH oxidase/ROS in pro-inflammatory mediators-induced airway and pulmonary diseases. Biochem. Pharmacol..

[18] Nadeem A., Siddiqui N., Alharbi N.O., Alharbi M.M., Imam F., Sayed-Ahmed M.M. (2014). Glutathione modulation during sensitization as well as challenge phase regulates airway reactivity and inflammation in mouse model of allergic asthma. Biochim..

[19] Zuo L., Otenbaker N.P., Rose B.A., Salisbury K.S. (2013). Molecular mechanisms of reactive oxygen species-related pulmonary inflammation and asthma. Mol. Immunol..

[20] Berger S.L. (2007). The complex language of chromatin regulation during transcription. Nature.

[21] Mitchell T., Chacko B., Ballinger S.W., Bailey S.M., Zhang J., Darley-Usmar V. (2013). Convergent mechanisms for dysregulation of mitochondrial quality control in metabolic disease: Implications for mitochondrial therapeutics. Biochem. Soc. Trans..

[22] Gasana J., Dillikar D., Mendy A., Forno E., Ramos Vieira E. (2012). Motor vehicle air pollution and asthma in children: A meta-analysis. Environ. Res..

[23] Miller R.L., Peden D.B. (2014). Environmental effects on immune responses in patients with atopy and asthma. J. Allergy Clin. Immunol..

[24] Custovic A., Marinho S., Simpson A. (2012). Gene–environment interactions in the development of asthma and atopy. Expert Rev. Respir. Med..

[25] Tezza G., Mazzei F., Boner A. (2013). Epigenetics of allergy. Early Hum. Dev..

[26] Rufo J.C., Madureira J., Fernandes E.O., Moreira A. (2016). Volatile organic compounds in asthma diagnosis: A systematic review and meta-analysis. Allergy.

[27] Pedreschi F., Mariotti M.S., Granby K. (2014). Current issues in dietary acrylamide: Formation, mitigation and risk assessment. J. Sci. Food Agric..

[28] Xu Y., Cui B., Ran R., Liu Y., Chen H., Kai G., Shi J. (2014). Risk assessment, formation, and mitigation of dietary acrylamide: Current status and future prospects. Food Chem. Toxicol..

[29] Friedman M. (2015). Acrylamide: Inhibition of formation in processed food and mitigation of toxicity in cells, animals, and humans. Food Funct..

[30] Semla M., Goc Z., Martiniakova M., Omelka R., Formicki G. (2017). Acrylamide: A common food toxin related to physiological functions and health. Physiol. Res..

[31] Hoffman S., Nolin J., McMillan D., Wouters E., Janssen-Heininger Y., Reynaert N. (2015). Thiol redox chemistry: Role of protein cysteine oxidation and altered redox homeostasis in allergic inflammation and asthma. J. Cell Biochem..

[32] Kirkham P., Rahman I. (2006). Oxidative stress in asthma and COPD: Antioxidants as a therapeutic strategy. Pharmacol. Ther..

[33] Andreyev A.Y., Kushnareva Y.E., Starkov A.A. (2005). Mitochondrial metabolism of reactive oxygen species. Biochem. (Mosc.).

[34] Ward J.P.T. (2006). Point: Hypoxic pulmonary vasoconstriction is mediated by increased production of reactive oxygen species. J. Appl. Physiol. (1985).

[35] Gureev A., Shaforostova E.A., Popov V., Starkov A. (2019). Methylene blue does not bypass Complex III antimycin block in mouse brain mitochondria. FEBS Lett..

[36] Hung C.-H., Yang S.-N., Kuo P.-L., Chu Y.-T., Chang H.-W., Wei W.-J., Huang S.-K., Jong Y.-J. (2010). Modulation of cytokine expression in human myeloid dendritic cells by environmental endocrine-disrupting chemicals involves epigenetic regulation. Environ. Health Perspect..

[37] Ojiaku C.A., Yoo E.J., Panettieri R.A. (2017). Transforming growth factor beta1 function in airway remodeling and hyperresponsiveness. The missing link?. Am. J. Respir. Cell Mol. Biol..

[38] Jaffer O.A., Carter A.B., Sanders P.N., Dibbern M.E., Winters C.J., Murthy S., Ryan A.J., Rokita A.G., Prasad A.M., Zabner J. (2015). Mitochondrial-targeted antioxidant therapy decreases transforming growth factor-beta-mediated collagen production in a murine asthma model. Am. J. Respir. Cell. Mol. Biol..

[39] Youle R.J., Narendra D.P. (2011). Mechanisms of mitophagy. Nat. Rev. Mol. Cell Biol..

[40] Patel A.S., Song J.W., Chu S.G., Mizumura K., Osorio J.C., Shi Y., El-Chemaly S., Lee C.G., Rosas I.O., Elias J.A. (2015). Epithelial cell mitochondrial dysfunction and PINK1 are induced by transforming growth factor- beta1 in pulmonary fibrosis. PLoS ONE.

[41] Larson-Casey J.L., Deshane J.S., Ryan A.J., Thannickal V.J., Carter A.B. (2016). Macrophage Akt1 kinase-mediated mitophagy modulates apoptosis resistance and pulmonary fibrosis. Immunity.

[42] Zhao Y., Guo Y., Jiang Y., Zhu X., Liu Y., Zhang X. (2017). Mitophagy regulates macrophage phenotype in diabetic nephropathy rats. Biochem. Biophys. Res. Commun..

[43] Messer J.S. (2017). The cellular autophagy/apoptosis checkpoint during inflammation. Cell. Mol. Life Sci..

[44] Hung C.H., Wang C.C., Suen J.L., Sheu C.C., Kuo C.H., Liao W.T., Yang Y.H., Wu C.C., Leung S.Y., Lai R.S. (2018). Altered pattern of monocyte differentiation and monocyte-derived TGF-beta1 in severe asthma. Sci. Rep..

[45] Trotta A.P., Chipuk J.E. (2017). Mitochondrial dynamics as regulators of cancer biology. Cell. Mol. Life Sci..

[46] Pietrocola F., Izzo V., Niso-Santano M., Vacchelli E., Galluzzi L., Maiuri M.C., Kroemer G. (2013). Regulation of autophagy by stress-responsive transcription factors. Semin. Cancer Biol..

[47] Sánchez-Sánchez A.M., Martin V., García-Santos G., Rodríguez-Blanco J., Casado-Zapico S., Suarez-Garnacho S., Antolín I., Rodriguez C. (2011). Intracellular redox state as determinant for melatonin antiproliferative vs cytotoxic effects in cancer cells. Free Radic. Res..

[48] Florea S.M., Blatter L.A. (2010). The role of mitochondria for the regulation of cardiac alternans. Front. Physiol..

[49] Lin Y.-C., Lin Y.-C., Huang M.-Y., Kuo P.-L., Wu C.-C., Lee M.-S., Hsieh C.-C., Kuo H.-F., Kuo C.-H., Tsai W.-C. (2017). Tumor necrosis factor-alpha inhibitors suppress CCL2 chemokine in monocytes via epigenetic modification. Mol. Immunol..

[50] Lee C.-H., Wu S.-B., Hong C.-H., Liao W.-T., Wu C.-Y., Chen G.-S., Wei Y.-H., Yu H.-S. (2011). Aberrant cell proliferation by enhanced mitochondrial biogenesis via mtTFA in arsenical skin cancers. Am. J. Pathol..

